# Bioinformatic analysis revealing mitotic spindle assembly regulated NDC80 and MAD2L1 as prognostic biomarkers in non-small cell lung cancer development

**DOI:** 10.1186/s12920-020-00762-5

**Published:** 2020-08-14

**Authors:** Rong Wei, Ziyue Wang, Yaping Zhang, Bin Wang, Ningning Shen, Li E, Xin Li, Lifang Shang, Yangwei Shang, Wenpeng Yan, Xiaoqin Zhang, Wenxia Ma, Chen Wang

**Affiliations:** Department of Pathology, The Second Hospital of ShanXi Medical University, No.382 WuYi Road, Tai Yuan City, 030000 ShanXi Province China

**Keywords:** Non-small cell lung cancer (NSCLC), Lung adenocarcinoma (LUAD), Squamous cell carcinoma (LUSC), GEO database, TCGA data, Biomarker

## Abstract

**Background:**

Lung cancer has been the leading cause of tumor related death, and 80% ~ 85% of it is non-small cell lung cancer (NSCLC). Even with the rising molecular targeted therapies, for example EGFR, ROS1 and ALK, the treatment is still challenging. The study is to identify credible responsible genes during the development of NSCLC using bioinformatic analysis, developing new prognostic biomarkers and potential gene targets to the disease.

**Methods:**

Firstly, three genes expression profiles GSE44077, GSE18842 and GSE33532 were picked from Gene Expression Omnibus (GEO) to analyze the genes with different expression level (GDEs) between NSCLC and normal lung samples, and the cellular location, molecular function and the biology pathways the GDEs enriched in were analyzed. Then, gene function modules of GDEs were explored based on the protein-protein interaction network (PPI), and the top module which contains most genes was identified, followed by containing genes annotation and survival analysis. Moreover, multivariate cox regression analysis was performed in addition to the Kaplan meier survival to narrow down the key genes scale. Further, the clinical pathological features of the picked key genes were explored using TCGA data.

**Results:**

Three GEO profiles shared a total of 664 GDEs, including 232 up-regulated and 432 down-regulated genes. Based on the GDEs PPI network, the top function module containing a total of 69 genes was identified, and 31 of 69 genes were mitotic cell cycle regulation related. And survival analysis of the 31 genes revealed that 17/31 genes statistical significantly related to NSCLC overall survival, including 4 spindle assembly checkpoints, namely NDC80, BUB1B, MAD2L1 and AURKA. Further, multivariate cox regression analysis identified NDC80 and MAD2L1 as independent prognostic indicators in lung adenocarcinoma (LUAD) and squamous cell carcinoma (LUSC) respectively. Interestingly, pearson correlation analysis indicated strong connection between the four genes NDC80, BUB1B, MAD2L1 and AURKA, and their clinical pathological features were addressed.

**Conclusions:**

Using bioinformatic analysis of GEO combined with TCGA data, we revealed two independent prognostic indicators in LUAD and LUSC respectively and analyzed their clinical features. However, more detailed experiments and clinical trials are needed to verify their drug targets role in clinical medical use.

## Background

Lung cancer has been a common malignant tumor worldwide [1], with the morbidity only second to prostatic cancer in male and breast cancer in female [2]. As for the mortality, lung cancer has been the top killer of cancer-related death both in male and female for decades [3, 4]. The other two cancer-related death that next to lung cancer are prostatic cancer and colorectal cancer in male, as well as breast cancer and colorectal cancer in female, the four cancer types taking up 45% of the whole malignant tumor related death roll. Meanwhile within the lung cancer, 80% ~ 85% is non-small cell lung cancer (NSCLC), including adenocarcinoma, squamous cell carcinoma and large cell carcinoma [2].

Besides the traditional surgery, chemotherapy and radiotherapy, targeted therapy is a newly developed clinical curative method in NSCLC involving tens genes, including EGFR, ALK, ROS1, BRAF, HER2, PIK3CA, RET and so on [5]. For instance, the discovery of the frequent mutation of EGFR in NSCLC especially lung adenocarcinoma in non-smoking female Asia patients leading to the development of generations EGFR-TKI (tyrosine kinase inhibitors) treatment, which has been showing effective results [6–8]. Additionally, the rearrangements of ALK, ROS1 and RET genes bring in the development of therapeutic TKI treatments, for instance crizotinib and lorlatinib [9, 10]. The overall disease responsive rate is reported to be as high as 55%, meanwhile the progression-free survival rate reaches 72% in NSCLC patients with ALK rearrangement [11, 12].

However, the currently available drug targets are lacking as opposed to the progressively developing cancer. Even with the rising molecular targeted therapies that shows promising treatment effects, the current situation for NSCLC clinical treatment is not promising. To understand more clear about the genetic information of NSCLC thus identifying potential prognostic biomarkers and new drug targeting genes is of great importance.

Recently, the development of high-throughput technologies, for instance protein chips, next generation sequencing and single cell sequencing bring in tremendous molecular data, which are publicly available, providing great chances for us to uncover novel genomic targets for therapeutic intervention [13, 14].

In the study, three cDNA expression profiles GSE44077 [15], GSE18842 [16] and GSE33532 [17] were firstly picked from Gene Expression Omnibus (GEO) based on their sample number to detect the genes with different expression level (GDEs) in NSCLC versus normal lung samples. Then, based on the protein-protein interacting (PPI) network of GDEs, GDEs function modules were analyzed and the top module containing most GDEs was picked, and all the containing genes were identified to evaluate the association with patients overall survival (OS) using KM plot online database and cox regression analysis. Moreover, the cellular component, molecular functions, signaling pathways and biological processes of the hub genes, namely the genes that were statistical significantly correlate with NSCLC OS would be analyzed, and their clinical pathological features would be evaluated using TCGA data. The results shall be useful for identifying new prognostic biomarkers and potential gene targets in clinical NSCLC treatment.

## Methods

### Data source: three cDNA profiles from GEO online database

From GEO online public database [18], three cDNA expression datasets GSE18842, GSE44077 and GSE33532 were picked based on the sample size (Only the profiles that contain at least 20 paired samples were considered). Within the 3 profiles, GSE18842 was based on GPL570[HG-U133_Plus_2] Affymetrix Human Genome U133 Plus 2.0 Array, containing 46 NSCLC cancer and 45 normal lung samples. And GSE44077 profile was based on GPL6244[HuGene-1_0-st] Affymetrix Human Gene 1.0 ST Array, including 55 cancer and 66 normal lung samples. And GSE33532 was based on GPL570[HG-U133_Plus_2] Affymetrix Human Genome U133 Plus 2.0 Array, covering 80 cancer and 20 normal samples.

### Unearth of the GDEs in NSCLC from normal lung samples

GEO2R tool [19] is provided pared with GEO data online, and in the study, it wad used to analyze the GDEs between NSCLC and normal lung samples. The criteria for GDEs identification were set as |log2FC| ≥1 and adjusted *P* value < 0.05.

### Pathway enrichment of GDEs revealed by GO and KEGG

Gene ontology analysis (GO) is effectively used to identify characteristic biological attributes of high-throughput genetic data. Meanwhile Kyoto Encyclopedia of Genes and Genomes (KEGG) is a collection of high throughput biological information covering genomes, cells, signaling pathways and so on, it is commonly used for annotation the lists of genes and interpretation of the network of signaling pathways involved. GO and KEGG analysis were performed using FUNRICH3.1 software [20] to reveal the functions enrichment of the GDEs shared in three GEO profiles, including their cellular components, molecular functions, biological processes and the signaling pathways they mainly enriched in.

### Construction of the PPI network of GDEs

STRING [21] is short for Search Tool for the Retrieval of interacting Genes, and it was used in the study to analyze the protein-protein interaction (PPI) of the GDEs uncovered by GEO2R. The analyzing criteria was set as confidence score ≥ 0.4 and maximum interactors number = 0.

### GDEs function module analysis based on PPI network

Molecular Complex Detection (MCODE) plug-in of Cytoscape3.6.0 software [22] was used to screen gene function modules based on GDEs PPI network, with the degree cut-off set as 2, node score cut off set as 0.2, the k-core equals 2, and max depth equals 100. Using MCODE analysis, we identified the top gene module (gene clusters sharing similar function) containing most GDEs, and GO and KEGG were further performed to annotate the genes and explore the signaling pathways of the gene modules.

### Survival analysis of module genes to identify key genes

Kaplan Meier plot [23] is an openly accessed online service for analyzing univariate overall survival correlation of multiple genes in various cancers including lung cancer. In the study, Kaplan Meier plot was firstly used to analyze the OS prognosis information of all the genes in the top module to screen for the genes that have statistical significant correlation with NSCLC patients survival.

And then multivariate COX regression analysis was performed using TCGA mRNA transcription data including 223 lung adenocarcinoma and 482 lung squamous cell carcinoma, which were downloaded from TCGA database [24] to identify the independent prognostic indicators from the univariate significant gene lists. Further, the genes’ association with clinical features were validated using lung adenocarcinoma and lung squamous cell carcinoma samples data provided on an online server UALCAN.

### Related signaling pathways and co-expression genes analysis

GEPIA [25] is a commonly used online service for analyzing certain genes expression differences between cancer and normal tissues in various tumor types and exploring the correlation between genes. In the study, we used GEPIA to analyze key genes’ (the genes that statistical significantly correlates with NSCLC OS) general expression in lung cancer comparing to normal lung samples and explore the genes that harbor similar expression with analyzed key genes.

## Results

### Identification of 664 GDEs shared by three GEO profiles

**Fig. 1:**
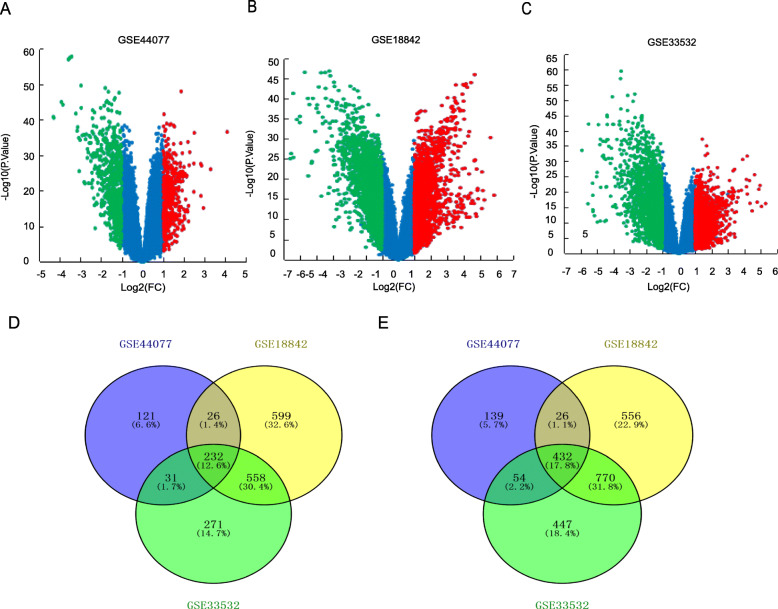
The GDEs analyzed by three GEO expression profiles. Up-regulated (red-colored) and down-regulated (green-colored) GDEs in NSCLC versus normal lung tissues analyzed based on GEO profiles **a** GSE44077, **b** GSE18842 and **c** GSE33532 respectively. 232 up-regulated GDEs and (E) 432 down-regulated GDEs were shared in three cDNA expression profiles

Three GEO cDNA profiles GSE44077, GSE18842 and GSE33532 were picked to analyze the GDEs in cancer vs. normal lung samples. And a whole of 1133, 4459 and 3775 GDEs including 691, 2505, 2351 down-regulated and 442, 1954, 1424 up-regulated genes were identified in GSE44077 (Fig. 1a), GSE18842 (Fig. 1b) and GSE33532 (Fig. 1c) respectively. Additionally, 432 down-regulated and 232 up-regulated GDEs were shared among the three GEO profiles showed by Venn diagram performance (Fig. 1d, e).

### Pathway enrichment analysis of shared GDEs by GO and KEGG

**Fig. 2:**
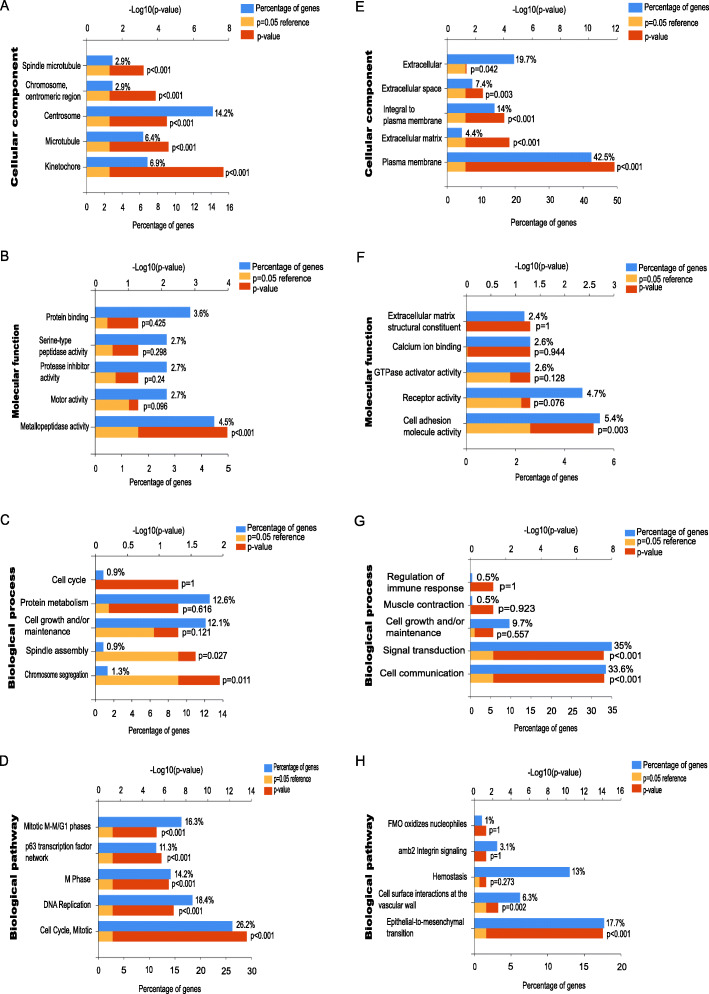
GDEs function analysis by GO and KEGG in NSCLC. The cellular components, **b** molecular functions, **c** biological processes, and **d** biological pathways the up-regulated GDEs were enriched in. The cellular components, **f** molecular functions, **g** biological processes, and **h** biological pathways the down-regulated GDEs were enriched in

To further understand the pathways 664 GDEs were mainly enriched in, GO and KEGG analysis were conducted. Interestingly, GO analysis showed that the cell components of 232 up-regulated GDEs were enriched in centrosome, microtubule and kinetochore (Fig. 2a), and the molecular function were focused on metallopeptidase activity (Fig. 2b). The biological process were mostly enriched in cell growth and maintenance, spindle assembly and chromosome segregation (Fig. 2c). Moreover, KEGG/biological pathway analysis showed the up-regulated GDEs were mostly involved in cell mitotic and DNA replication (Fig. 2d). Three of the four aspects including genes cell component, signaling pathways and biological process suggested the orientation of cell cycle mitotic process, indicating the potential value of cell division process in cancer targeting treatment.

Meanwhile, as for the 432 down regulated GDEs, the cell components were primary focused on cellular plasma membrane (Fig. 2e), the molecular function were enriched in receptor activity and cell adhesion molecular activity (Fig. 2f), and the biological process were mainly enriched in signal transduction and cell communication (Fig. 2g). Additionally, KEGG/biological pathway analysis showed the down-regulated GDEs were mostly participated in hemostasis, cell surface interaction at vascular walls and Epithelial to Mesenchymal transition (EMT) (Fig. 2h).

### Function module analysis based on PPI network

**Fig. 3:**
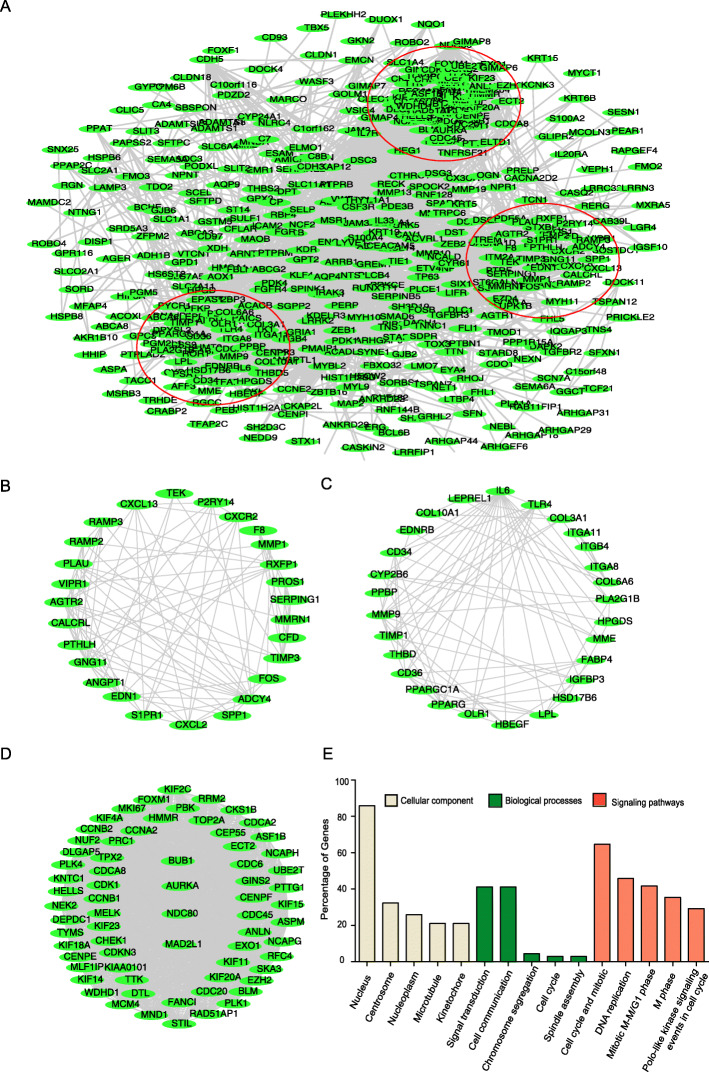
GDEs gene function modules analysis based on PPI network. The PPI network of 664 GDEs, and three top gene modules analyzed based on the network, each red circle represents one gene module. **d** Three gene modules containing **b** 27, **c** 28 and **d** 69 GDEs respectively. **e** GO and KEGG analysis reveal the basis functions including cellular components, biological processes and signaling pathways the 69 genes in top module mainly enriched in

To identify the potential responsible genes in NSCLC development, the PPI network of 664 GDEs was constructed with STRING, and the function modules of the GDEs were analyzed. Based on the PPI, top three gene modules were identified containing 69, 27 and 28 genes respectively (Fig. 3a), and these three modules were named as Gene Cluster1 (Fig. 3d), 2 (Fig. 3b) and 3 (Fig. 3c) accordingly.

GO and KEGG result revealed that most of the Cluster 1 genes were enriched in the cell cycle (31/69), DNA replication (22/69) and Mitotic M-M/G1 (20/69) related signaling (Fig. 3e). All the signaling that Cluster1 genes enriched in were sorted in descending order based on the gene counts and FDR value (Table 1). We primarily focused on the top cell cycle regulation related module which matches most GDEs in the network, and we further perform survival analysis on all the 31 genes.

**Table 1:** Signaling pathways that Cluster 1 genes mainly enriched in

pathways	count	***P*** value	Related GDEs in the network
Cell Cycle, Mitotic	31	2.76403E-29	CENPE; KNTC1; KIF18A; TOP2A; MAD2L1; CCNB2; CKS1B; MCM4; CCNB1; PTTG1; NUF2; CDK1; AURKA; PLK1; TYMS; CCNA2; CDC20; NDC80; BUB1; CDC6; GINS2; CDC45; PLK4; CDCA8; KIF20A; RRM2; KIF2C; RFC4; KIF23; CENPF; NEK2;
DNA Replication	22	1.71367E-18	CENPE; KNTC1; KIF18A; MAD2L1; MCM4; NUF2; CDK1; PLK1; CDC20; NDC80; BUB1; CDC6; GINS2; CDC45; PLK4; CDCA8; KIF20A; KIF2C; RFC4; KIF23; CENPF; NEK2;
Mitotic M-M/G1 phases	20	1.48601E-16	CENPE; KNTC1; KIF18A; MAD2L1; MCM4; NUF2; CDK1; PLK1; CDC20; NDC80; BUB1; CDC6; CDC45; PLK4; CDCA8; KIF20A; KIF2C; KIF23; CENPF; NEK2;
M Phase	17	5.76137E-16	CENPE; KNTC1; KIF18A; MAD2L1; NUF2; CDK1; PLK1; CDC20; NDC80; BUB1; PLK4; CDCA8; KIF20A; KIF2C; KIF23; CENPF; NEK2;
Polo-like kinase signaling events in the cell cycle	14	2.77681E-14	CENPE; CCNB1; CDK1; AURKA; TPX2; ECT2; PLK1; PRC1; CDC20; NDC80; BUB1; PLK4; DLGAP5; KIF20A;
PLK1 signaling events	13	3.83583E-13	CENPE; CCNB1; CDK1; AURKA; TPX2; ECT2; PLK1; PRC1; CDC20; NDC80; BUB1; DLGAP5; KIF20A;
Mitotic Prometaphase	12	5.11023E-12	CENPE; KNTC1; KIF18A; MAD2L1; NUF2; PLK1; CDC20; NDC80; BUB1; CDCA8; KIF2C; CENPF;
Cell Cycle Checkpoints	11	1.32039E-08	MAD2L1; CCNB2; MCM4; NDC80; CCNB1; CDK1; CDC20; CDC6; CDC45; RFC4; CHEK1;
Signaling by Aurora kinases	11	1.04137E-10	NCAPH; NCAPG; AURKA; TPX2; NDC80; BUB1; CDCA8; DLGAP5; KIF20A; KIF2C; KIF23;

Only pathways containing over 10 GDEs were listed

### Survival analysis of cluster 1 module genes

**Fig. 4:**
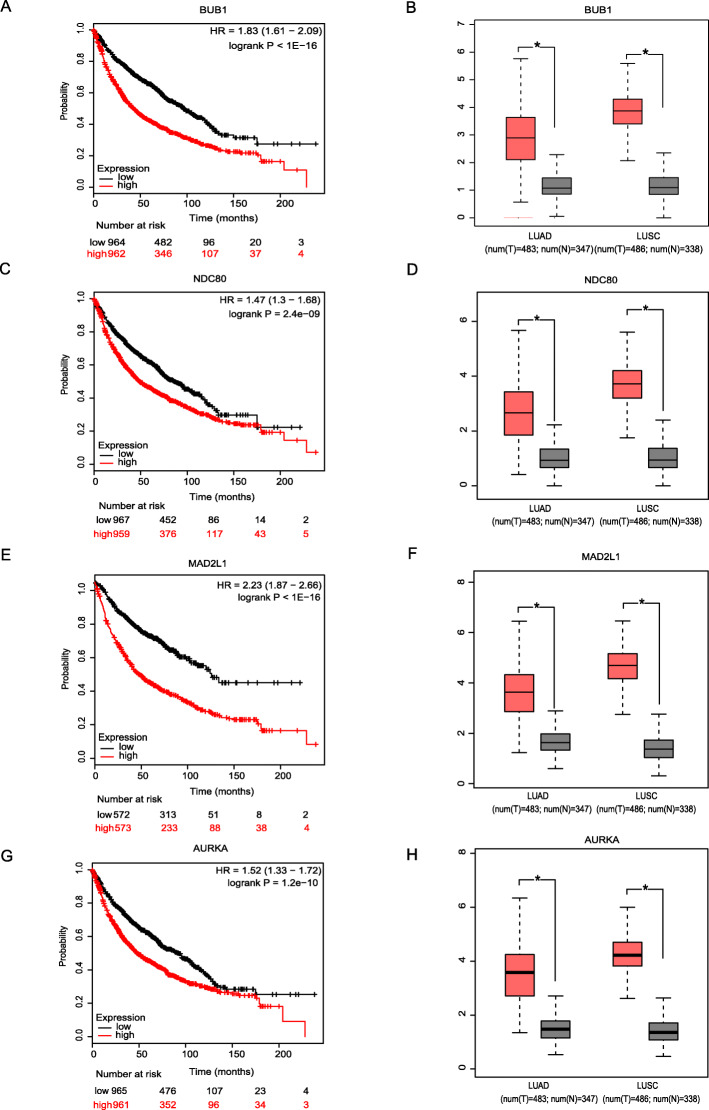
Os prognosis and expression information of 4 spindle assembly checkpoints genes. Overall survival value of **a** BUB1, **c** NDC80, **e** MAD2L1 and **g** AURKA in NSCLC; Expression level of **b** BUB1, **d** NDC80, **f** MAD2L1 and **h** AURKA in NSCLC cancer versus normal lung tissues, including adenocarcinoma (left column) squamous cancer (right column). * *P* < 0.05

Univariate Kaplan Meier plot overall survival analysis of 31 cell cycle regulation genes in Gene Cluster 1 showed that 17 out of 31 genes statistical significantly correlates with patients overall survival, including 4 spindle assembly checkpoints BUB1 (Fig. 4a), NDC80 (Fig. 4c), MAD2L1 (Fig. 4e), and AURKA (Fig. 4g). And GEPIA was then used to validate genes’ gaped expression in NSCLC versus normal lung samples, and the results showed the gain of expression of all four genes in cancer comparing to normal samples (Fig. 4b, d, f, h).

Further, multivariate cox regression analysis showed that patients age, p-stage, M status and NDC80 expression work as independent prognostic indicators in adenocarcinoma (Table 2), meanwhile, T stage, M status and MAD2L1 expression work as an independent indicators in squamous cell carcinoma (Table 3).

**Table 2:** Multivariate cox regression analysis on LUAD overall survival

Variables	Lung adenocarcinoma		
	Hazard ratio	95% CI	*P* value
Age			
< 60 years vs ≥60 years	1.034	1.006 ~ 1.064	0.018
Stage			
I vs II vs III vs IV	1.554	1.213 ~ 1.99	0.001
M			
M_o_ vs M_1_	2.321	1.172 ~ 4.069	0.032
NDC80 expression			
< median vs > median	2.480	1.375 ~ 4.472	0.003

**Table 3:** Multivariate cox regression analysis on LUSC overall survival

Variables	Lung squamous cell carcinoma		
	Hazard ratio	95% CI	*P* value
T			
T1 vs T2 vs T3 vs T4	1.305	1.085 ~ 1.569	0.005
M			
M_o_ vs M_1_	1.701	1.172 ~ 2.469	0.005
MAD2L1 expression			
< median vs > median	0.753	0.567 ~ 0.999	0.045

### NDC80 and MAD2L1 association with NSCLC clinical features

To explore the clinical association between NDC80 and MAD2L1 expression with LUAD and LUSC clinical features, we used two methods. Firstly, the clinical information of 482 lung squamous cell carcinoma (Detailed in Table S1) and 223 adenocarcinoma cases (Table S2) were downloaded from TCGA data (same information being used for COX regression analysis), and the results showed that NDC80 expression statistical significantly associates with LUAD patients age, smoking, and stage in adenocarcinoma, the gene tends to express higher in younger (< 60 years), smoker and higher stage patients (Table 4). And MAD2L1 expression statistical significantly associates with LUSC lympho node and distant metastasis, the expression was higher in patients with lympho node metastasis but no distant metastasis (Table 5).

**Table 4:** The association between NDC80 and LUAD clinical pathological features

parameters	NDC80		*P* value
	–	+	
Gender			*P* = 0.209
male	45 (45.9%)	53 (54.1%)	
female	68 (54.4%)	57 (45.6%)	
Age			*P* = 0.027
< 60 years old	24 (39.3%)	37 (60.7%)	
≥ 60 years old	82 (56.2%)	64 (43.8%)	
Smoke			*P* = 0.002
no	23 (65.7%)	12 (34.3%)	
Current smoker	15 (32.6%)	31 (67.4%)	
Smoker < 15 years	31 (44.9%)	38 (55.1%)	
Smoker ≥15 years	39 (59.1%)	27 (40.9%)	
Stage			*P* = 0.032
I	66 (57.4%)	49 (42.6%)	
II	23 (48.9%)	24 (51.1%)	
III	15 (31.9%)	32 (68.2%)	
IV	5 (55.6%)	4 (44.4%)	
T			*P* = 0.199
I	37 (61.7%)	23 (38.3%)	
II	63 (47.7%)	68 (52.3%)	
III	7 (41.2%)	10 (58.8%)	
IV	6 (40.0%)	9 (60.0%)	
N			*P* = 0.053
-	69 (56.6%)	53 (43.4%)	
+	44 (43.6%)	57 (56.4%)	
M			*P* = 0.497
-	81 (50.9%)	78 (49.1%)	
+	27 (45.8%)	31 (54.2%)

**Table 5:** The association between MAD2L1 and LUSC clinical pathological features

parameters	MAD2L1		*P* value
	–	+	
Gender			*P* = 0.097
male	170 (47.8%)	186 (52.2%)	
female	71 (56.3%)	55 (43.7%)	
Age			*P* = 0.100
< 60 years old	44 (42.7%)	59 (57.3%)	
≥ 60 years old	192 (51.9%)	178 (48.1%)	
Smoke			*P* = 0.973
no	131 (50.6%)	128 (49.4%)	
Smoker < 15 years	28 (50.0%)	28 (50.0%)	
Smoker ≥15 years	82 (49.4%)	84 (50.6%)	
Stage			*P* = 0.062
I	132 (56.2%)	103 (43.8%)	
II	70 (44.6%)	87 (55.4%)	
III	35 (42.7%)	47 (57.3%)	
IV	4 (57.1%)	3 (42.9%)	
T			*P* = 0.314
I	60 (56.1%)	47 (43.9%)	
II	131 (46.5%)	151 (53.5%)	
III	38 (54.3%)	32 (45.7%)	
IV	12 (52.2%)	11 (47.8%)	
N			*P* = 0.001
-	171 (55.9%)	135 (44.1%)	
+	70 (39.8%)	106 (60.2%)	
M			*P* = 0.036
-	188 (48.0%)	204 (52.0%)	
+	52 (60.5%)	34 (39.5%)	

**Fig. 5:**
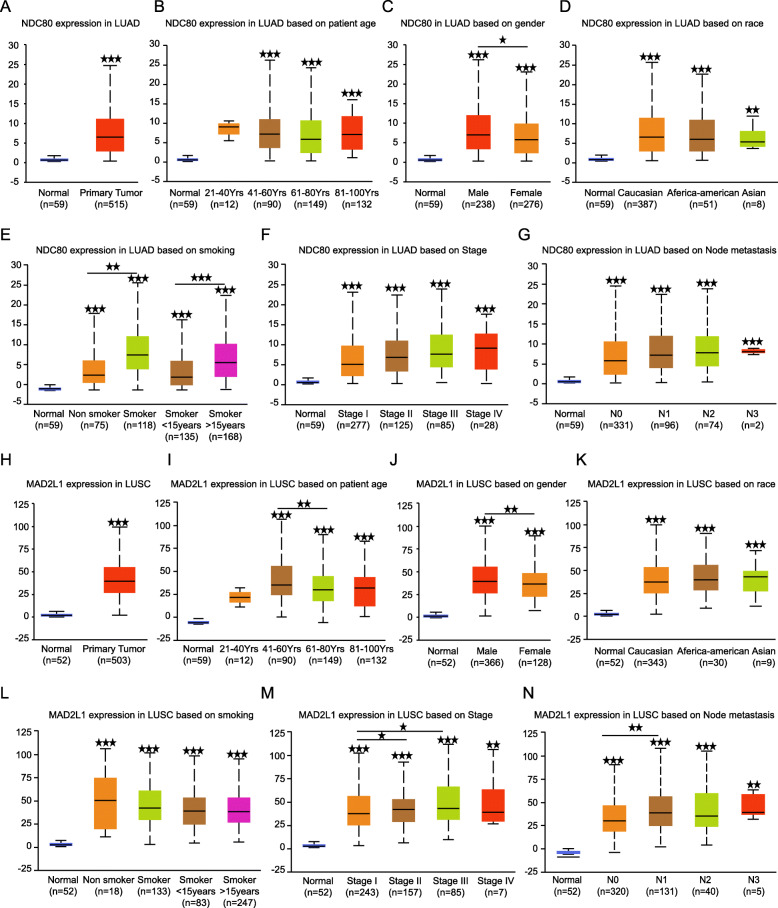
The relationship between NDC80, MAD2L1 expression and LUAD, LUSC clinical parameters. Relative NDC80 expression in LUAD. And the association between NDC80 expression and adenocarcinoma **b** patients age, **c** gender, **d** race, **e** smoking status, **f** tumor stage and **g** lymph node metastasis. h Relative MAD2L1 expression in lung squamous cell carcinoma. And the association between MAD2L1 expression and squamous cell carcinoma **i** patients age, **j** gender, **k** race, **l** smoking status, **m** tumor stage and **n** lymph node metastasis.* p < 0.05, **p < 0.01, ***p < 0.001. (The first layer * which is right above the error bar representing comparison to normal group,and the above layers * which were above a secondary line represent the comparison between corresponding groups that were covered by the line)

Secondly, an online analysis service Ualcan which is also based on TCGA data was also used for data exploration (Fig. 5a-n), the result also revealed that NDC80 expresses higher in smoker than non smokers and the expression increases as the smoking years lasting longer (Fig. 5d), and NDC80 tends to be higher in cases with lympho node netastasis (Fig. 5g). Interestingly, bigger sample number also yields the discovery that both NDC80 (Fig. 5c) and MAD2LI (Fig. 5j) express higher in male than female patients, hypothetically, the gender association might be related to the fact that most smokers were man rather than woman.

### NDC80 and MAD2L1 centered signaling pathways

**Fig. 6:**
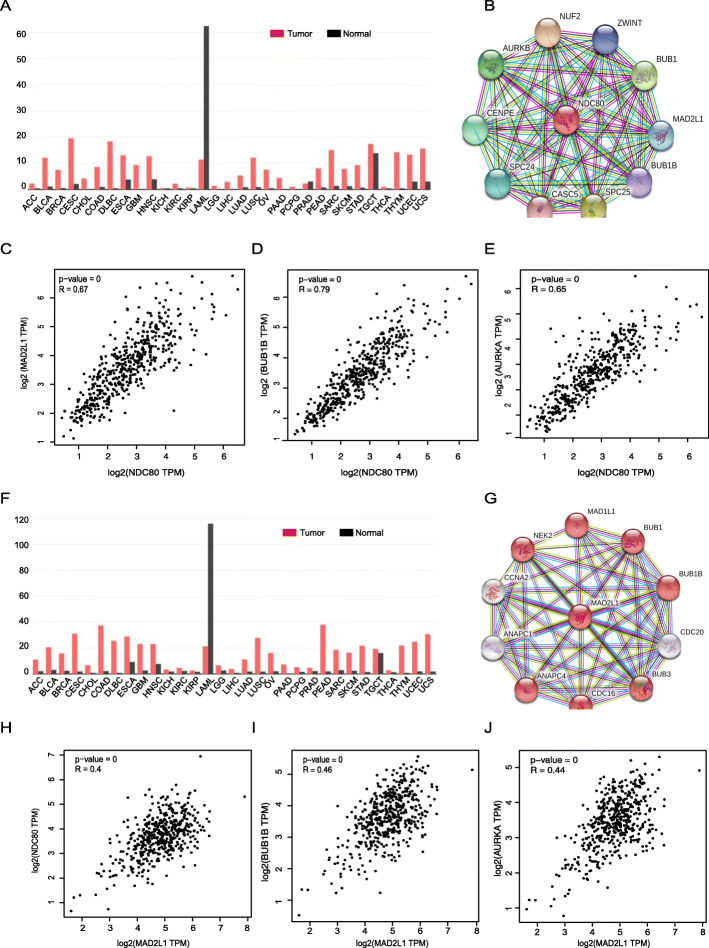
NDC80 and MAD2L1 related signaling analysis. Expression of NDC80 in various human cancers revealed by GEPIA. NDC80 centered PPI network representing the genes most related to NDC80. **e** Correlation between NDC80 and **c** MAD2L1, **d** BUB1B and **e** AURKA in LUAD revealed by GEPIA (R = 0.67, 0.79, 0.65 respectively). **f** Expression of MAD2L1 in various human cancers revealed by GEPIA. **g** MAD2L1 centered PPI network representing the genes most related to MAD2L1. **h-j** Correlation between MAD2L1 and **h** NDC80, **i** BUB1B and **j** AURKA in LUSC revealed by GEPIA (R = 0.40, 0.46, 0.44 respectively)

The expression profile of NDC80 and MAD2L1 was analyzed in various tumors using GEPIA and we discovered that both NDC80 and MAD2L1 were broad-spectrum up-regulated in multiple human tumors including lung adenocarcinoma and lung squamous cell carcinoma (Fig. 6a, f).

To understand the potential functions of NDC80 and MAD2L1, we performed GO and KEGG to analyze the biological processes the genes mainly participate in and the signaling pathways they involve. The result revealed an really interesting fact that even in different sub types of lung cancer (LUAD and LUSC), NDC80 and MAD2L1 shared biological functions. Both NDC80 (Table 6) and MAD2L1 (Table 7) were primarily focused on mitotic cell cycle regulation related processes, for instance cell division, chromosome segregation and spindle assembly regulating signaling.

**Table 6:** Go analysis revealing biological processes centered on NDC80

Description	Gene counts	Background gene counts	FDR	Matching proteins in the network
Cell division	11	483	7.33E-16	AURKB,BUB1,BUB1B,CASC5,NUF2,CENPE,MAD2L1,NDC80,SPC24,SPC25,ZWINT
Chromosome segregation	10	253	7.33E-16	AURKB,BUB1,BUB1B,CASC5,CENPE, MAD2L1,NDC80,NUF2,SPC25,ZWINT
Sister chromatid segregation	7	123	1.31E-11	AURKB,BUB1,BUB1B,CENPE,MAD2L1, NDC80,ZWINT
Spindle attachment to kinetochore	4	20	1.32E-08	AURKB,CASC5,CENPE,NDC80
Spindle checkpoint	4	23	2.07E-08	AURKB,BUB1,BUB1B,MAD2L1

**Table 7:** Go analysis revealing biological processes centered on MAD2L1

Description	Gene counts	Background gene counts	FDR	Matching proteins in the network
Cell division	10	483	1.09E-15	ANAPC1,ANAPC4,BUB1,BUB1B,BUB3, CDC16,CDC20,MAD1L1,MAD2L1,NEK2
Nuclear division regulation	9	184	9.05E-15	ANAPC4,BUB1,NEK2,BUB1B,BUB3, CDC16,CDC20,MAD1L1,MAD2L1
Chromosome organization	8	999	6.17E-08	BUB1,BUB1B,BUB3,CCNA2,CDC20, MAD1L1,MAD2L1,NEK2
anaphase-promoting complex-dependent catabolic process	7	35	9.05E-15	ANAPC1,ANAPC4,BUB1B,BUB3,CDC16,CDC20,MAD2L1
Mitotic spindle assembly checkpoint	5	21	4.46E-11	BUB1,BUB1B,BUB3,MAD1L1,MAD2L1

Moreover, NDC80 and MAD2L1 centered PPI network showed a similar result that the genes NDC80 (Fig. 6b) and MAD2L1 (Fig. 6g) related were both cell cycle regulation involved including BUB1B and AURKA. GEPIA analysis confirmed the correlation between NDC80 and MAD2L1, BUB1B, AURKA in both LUAD (Fig. 6c-e) and LUSC (Fig. 6h-j).

Considering that great proportion of current chemotherapy drugs are developed based on their association with cell mitosis cycle, the correlation between NDC80, MAD2L1 and cell division process indicate the potential value these genes working as two other chemotherapy drug targets. However, more experiments and clinical trials will be needed to validate the hypothesis.

## Discussion

Lung cancer has been the top killer among various malignant tumors worldwide, with the morbidity only second to prostatic cancer in male and breast cancer in female. Within the lung cancer, 80% ~ 85% is NSCLC. Even with the rising of molecular targeted therapies, including EGFR, BRAF, C-MET, ALK, ROS1, RET and so on, the outcome of the disease is still not promising. The study is conducted to explore new potential biomarkers and gene targets by bioinformatic analyzing.

From the online open-access GEO databases, three cDNA expression profiles GSE44077, GSE18842 and GSE33532 containing a total of of 181 NSCLC cancer and 131 normal lung samples were picked, and the GDEs between cancer versus normal tissues were then analyzed, and we discoved that 664 genes were differently expressed in three cDNA profiles, including 232 up-regulated and 432 down-regulated genes.

Then, we performed GO and KEGG analysis to annotate the 664 GDEs, and the results showed that the cell component that the 432 down-regulated genes mainly enriched in were plasma membrane, the biological processes the genes focused on were signal transduction and cell communication. The molecular functions that genes enriched in were receptor activity and cell adhesion molecular activity. Meanwhile, the biological pathways that down-regulated GDEs mostly enriched in were hemostasis and cell surface interaction at vascular walls.

To provoke our interests, three out of the four aspects the 232 up-regulated GDEs, including their cell growth and maintenance, spindle assembly and chromosome segregation enriched biological process, centrosome, microtubule and kinetochore centralized cell components and cell cycle/mitotic and DNA replication focused biological pathways point to the orientation of cell cycle mitotic process.

On top of it, the function modules analysis of GDEs revealed that most of the top module genes were also cell cycle regulation related. Overall survival analysis showed 17/31 of the top module genes statistical significantly correlate with NSCLC OS including four spindle assemble checkpoints NDC80, BUB1B, MAD2L1 and AURKA. Multivariate COX regression analysis supported NDC80 and MAD2L1 working as independent prognostic indicators in LUAD and LUSC respectively. Clinical features association analysis showed that NDC80 tends to expresses higher in younger (< 60 years) LUAD patients who smoke. And MAD2L1 usually expresses higher in LUSC patients with lympho node metastasis. Moreover, NDC80 and MAD2L1 centered biological processes and signaling pathways also highly support their involvement in the cell cycle regulation.

In fact, cell cycle regulators have been strongly implicated in the progression of various tumors [26, 27], and disruption of cell cycle pathways including spindle assembly has been one of the main focus of current development of chemotherapy drugs [28–30], for instance taxol and colchicine, which disrupts the microtubule polymerization dynamics, leading to inordinate spindle function and eventually cell death [31–34].

The over expression of multiple spindle checkpoints is revealing another potential microtubule-targeted strategy, the direct attack to spindle assemble checkpoint function, to arrest the cell cycle process in the prometaphase, thus leading to mitotic catastrophe and eventually cancer cell death.

NDC80, which is short for nuclear division cycle 80, is one of the proteins of outer kinetochore. It forms a heterotetramer complex with proteins SPC24, SPC25 and NUF2, and the complex has been known to involve in spindle assembly checkpoint signaling, detecting the unaligned chromosomes to assure the correct segregation of chromosomes. Aberrant expression of NDC80 has been reported in several other tumors [35–39], for instance osteosarcoma, hepatocellulcar carcinoma, colorectal cancer and breast cancer, indicating its potential as a newly bio target.

MAD2L1, short for mitosis arrest-deficient 2 like 1 protein, is also functioning as a spindle assembly checkpoint that assures the properly alignment of chromosomes at the metaphase plate during cell division. Despite the barely known signaling pathways it participated in, MAD2L1 is shown to interact with CDC20 and BUB1B [40, 41], and correlate with aberrant development of salivary duct carcinoma [42].

BUB1, which is encoded by BUB1B, has been known as a checkpoint for proper chromosome segregation, the abnormal expression of BUB1 has been reported to associate with poor survival and metastasis in various tumors including colorectal cancer, gastric cancer, bladder cancer, hepatocellular carcinoma and so on [35, 43–45]. In the study, using bioinformatic analysis, we confirmed the correlation between over expression of BUB1B and poor survival of NSCLC patients.

Aurora kinase A (AURKA) belongs on a family of serine/threonine kinases containing other two family members aurora kinase B and kinase C. The family members are known to have highly conserved genetic domains and shown to play vital roles in mitosis. As a serine/threonine kinases, AURKA activity peaks during the G2/M phase transition phase in the cell cycle, and associated with the regulation of spindle stability. Aurora A dysregulation has been associated with high occurrence of various cancers, for example breast, prostate, bladder, colorectal, gastric, ovarian, esophagus and pancreatic cancers. High expression of AURKA commonly correlates with advanced development and poor prognosis of cancers [46–48]. Osimertinib and rociletinib, two anti-cancer drugs for lung cancer, work by shutting off mutant EGFR [49], which initially kills cancerous tumors, but the tumors rewire and activate Aurora kinase A, becoming cancerous growths again [50, 51]. A recent study shows that to target both EGFR and Aurora shall prevents return of drug resistant tumors [52–54].

Further clinical validation of the tumor promoter and worse prognosis predictor function of NDC80 and MAD2L1 in local LUAD and LUSC patients as well as the genes’ relation with BUB1B and AURKA is on our way. More experimental investigations are needed to understand the detailed molecular signaling mechanism behind the cell cycle related genes regulation on NSCLC development.

## Conclusion

In conclusion, 664 GDEs between NSCLC and normal lung tissues were explored using bioinformatic analysis, and the cellular components, molecular functions, biological processes and the signaling they mainly enriched in were also revealed. Two spindle assembly checkpoints NDC80 and MAD2L1 were showed to correlate with LUAD and LUSC OS respectively. These bioinformation shall provide clues for the further unearth of new biomarkers and potential bio-targets in NSCLC.

## Supplementary information

**Additional file 1.** Supplementary Table 1 The TCGA patients barcode for 482 LUSC samples.

**Additional file 2.** Supplementary Table 2 The TCGA patients barcode for 223 LUAD patients samples.

## Data Availability

In the study, different web-based dastsets were used for data analysis. The web links to all the original data sources were listed as below: Three cDNA expression files GSE44077 (based on GPL6244[HuGene-1_0-st] Affymetrix Human Gene 1.0 ST Array. Web link: https://www.ncbi.nlm.nih.gov/geo/query/acc.cgi?acc=GSE44077), GSE18842 (based on GPL570[HG-U133_Plus_2] Affymetrix Human Genome U133 Plus 2.0 Array. Web link: https://www.ncbi.nlm.nih.gov/geo/query/acc.cgi?acc=GSE18842) and GSE33532 (based on GPL570[HG-U133_Plus_2] Affymetrix Human Genome U133 Plus 2.0 Array. Web link: https://www.ncbi.nlm.nih.gov/geo/query/acc.cgi?acc=GSE33532) were downloaded from Gene Expression Omnibus (GEO). And during the survival analysis of genes, 223 lung adenocarcinoma and 482 lung squamous cell carcinoma data were obtained from The Cancer Genome Atlas Program (TCGA) (Detailed in Supplementary Table 1 and Supplementary Table 2), meanwhile another analysis was conducted based on UALCAN (an interactive web resource for analyzing cancer transcriptome data. Web link: http://ualcan.path.uab.edu/analysis.html) provided lung adenocarcinoma and squamous cell carcinoma data. All data generated from the analysis process of this study are available from the corresponding author on reasonable request.

## Abbreviations

NSCLC: Non-Small Cell Lung Cancer
LUAD: Lung adenocarcinoma
LUSC: Lung squamous cell carcinoma
GEO: Gene Expression Omnibus
GDEs: Genes with different expression level
GO: Gene ontology
OS: Overall survival rate
KEGG: Kyoto Encyclopedia of Gene and Genome
PPI: Protein-protein interaction network
MCODE: Molecular Complex Detection
STRING: Search Tool for Retrieval of interacting Genes
EMT: Epithelial to Mesenchymal transition
