# Supplementary material for: Bioinformatic analysis revealing mitotic spindle assembly regulated NDC80 and MAD2L1 as prognostic biomarkers in non-small cell lung cancer development
Source: BMC Med Genomics. 2020 Aug 14;13:112. doi: 10.1186/s12920-020-00762-5 (PMC7437940; doi:10.1186/s12920-020-00762-5)
Supplement: Supplementary file 1 — Additional file 1. Supplementary Table 1 The TCGA patients barcode for 482 LUSC samples. [file 12920_2020_762_MOESM1_ESM.docx]

**Supplementary Table 1 The TCGA patients barcode for 482 LUSC samples**

| TCGA-18-3406 | TCGA-18-3419 | TCGA-21-5784 | TCGA-22-5479 | TCGA-33-4587 | TCGA-37-5819 |
| --- | --- | --- | --- | --- | --- |
| TCGA-18-3407 | TCGA-18-3421 | TCGA-21-5786 | TCGA-22-5480 | TCGA-33-4589 | TCGA-37-A5EL |
| TCGA-18-3408 | TCGA-18-4083 | TCGA-21-5787 | TCGA-22-5481 | TCGA-33-6737 | TCGA-37-A5EM |
| TCGA-18-3409 | TCGA-18-4086 | TCGA-21-A5DI | TCGA-22-5482 | TCGA-33-6738 | TCGA-37-A5EN |
| TCGA-18-3410 | TCGA-18-4721 | TCGA-22-0940 | TCGA-22-5483 | TCGA-33-A4WN | TCGA-39-5011 |
| TCGA-18-3411 | TCGA-18-5592 | TCGA-22-0944 | TCGA-22-5485 | TCGA-33-A5GW | TCGA-39-5016 |
| TCGA-18-3412 | TCGA-18-5595 | TCGA-22-1000 | TCGA-22-5489 | TCGA-33-AAS8 | TCGA-39-5019 |
| TCGA-18-3414 | TCGA-21-1070 | TCGA-22-1002 | TCGA-22-5491 | TCGA-33-AASB | TCGA-39-5021 |
| TCGA-18-3415 | TCGA-21-1071 | TCGA-22-1005 | TCGA-22-5492 | TCGA-33-AASD | TCGA-39-5022 |
| TCGA-18-3416 | TCGA-21-1072 | TCGA-22-1011 | TCGA-22-A5C4 | TCGA-33-AASI | TCGA-39-5024 |
| TCGA-18-3417 | TCGA-21-1075 | TCGA-22-1012 | TCGA-33-4532 | TCGA-33-AASJ | TCGA-39-5027 |
| TCGA-56-8622 | TCGA-21-1076 | TCGA-22-1016 | TCGA-33-4533 | TCGA-33-AASL | TCGA-39-5028 |
| TCGA-56-8623 | TCGA-21-1077 | TCGA-22-1017 | TCGA-33-4538 | TCGA-34-2596 | TCGA-39-5029 |
| TCGA-56-8624 | TCGA-21-1078 | TCGA-22-4591 | TCGA-33-4547 | TCGA-34-2600 | TCGA-39-5030 |
| TCGA-56-8625 | TCGA-21-1079 | TCGA-22-4593 | TCGA-33-4566 | TCGA-34-2604 | TCGA-39-5031 |
| TCGA-56-8626 | TCGA-21-1080 | TCGA-22-4594 | TCGA-33-4582 | TCGA-34-2605 | TCGA-39-5034 |
| TCGA-56-8628 | TCGA-21-1081 | TCGA-22-4595 | TCGA-33-4583 | TCGA-34-2608 | TCGA-39-5035 |
| TCGA-56-8629 | TCGA-21-1082 | TCGA-22-4596 | TCGA-33-4586 | TCGA-34-2609 | TCGA-39-5036 |
| TCGA-56-A49D | TCGA-21-1083 | TCGA-22-4599 | TCGA-37-4133 | TCGA-34-5231 | TCGA-39-5037 |
| TCGA-56-A4BW | TCGA-21-5782 | TCGA-22-4601 | TCGA-37-4135 | TCGA-34-5232 | TCGA-39-5039 |
| TCGA-56-A4BX | TCGA-21-5783 | TCGA-22-4604 | TCGA-37-4141 | TCGA-34-5234 | TCGA-39-5040 |
| TCGA-56-A4BY | TCGA-60-2695 | TCGA-22-4605 | TCGA-43-A474 | TCGA-34-5236 | TCGA-43-2576 |
| TCGA-56-A4ZJ | TCGA-60-2696 | TCGA-22-4607 | TCGA-43-A475 | TCGA-34-5239 | TCGA-43-2578 |
| TCGA-56-A4ZK | TCGA-60-2697 | TCGA-22-4609 | TCGA-43-A56U | TCGA-34-5240 | TCGA-43-2581 |
| TCGA-56-A5DR | TCGA-60-2698 | TCGA-22-4613 | TCGA-56-8309 | TCGA-34-5241 | TCGA-43-3394 |
| TCGA-56-A5DS | TCGA-60-2703 | TCGA-22-5471 | TCGA-56-8503 | TCGA-34-5927 | TCGA-43-3920 |
| TCGA-56-A62T | TCGA-60-2704 | TCGA-22-5472 | TCGA-56-8504 | TCGA-34-5928 | TCGA-43-5668 |
| TCGA-58-8386 | TCGA-60-2706 | TCGA-22-5473 | TCGA-63-5131 | TCGA-34-5929 | TCGA-43-5670 |
| TCGA-58-8387 | TCGA-60-2707 | TCGA-22-5474 | TCGA-63-6202 | TCGA-34-7107 | TCGA-43-6143 |
| TCGA-58-8388 | TCGA-60-2708 | TCGA-22-5477 | TCGA-63-7020 | TCGA-34-8454 | TCGA-43-6647 |
| TCGA-58-8390 | TCGA-60-2709 | TCGA-22-5478 | TCGA-63-7021 | TCGA-34-8455 | TCGA-43-6770 |
| TCGA-58-8391 | TCGA-60-2710 | TCGA-60-2720 | TCGA-63-7022 | TCGA-34-8456 | TCGA-43-6771 |
| TCGA-58-8392 | TCGA-60-2711 | TCGA-60-2721 | TCGA-63-7023 | TCGA-34-A5IX | TCGA-43-6773 |
| TCGA-58-8393 | TCGA-60-2712 | TCGA-60-2722 | TCGA-63-A5M9 | TCGA-37-3783 | TCGA-43-7656 |
| TCGA-58-A46J | TCGA-60-2713 | TCGA-60-2723 | TCGA-63-A5MB | TCGA-37-3789 | TCGA-43-7657 |
| TCGA-58-A46K | TCGA-60-2714 | TCGA-60-2724 | TCGA-63-A5MG | TCGA-37-3792 | TCGA-43-7658 |
| TCGA-58-A46L | TCGA-60-2715 | TCGA-60-2725 | TCGA-63-A5MH | TCGA-37-4129 | TCGA-43-8115 |
| TCGA-58-A46M | TCGA-60-2716 | TCGA-60-2726 | TCGA-63-A5MI | TCGA-37-4130 | TCGA-43-8116 |
| TCGA-58-A46N | TCGA-60-2719 | TCGA-63-5128 | TCGA-63-A5MJ | TCGA-37-4132 | TCGA-43-8118 |
| TCGA-66-2755 | TCGA-63-A5ML | TCGA-77-6842 | TCGA-77-A5GF | TCGA-85-A4PA | TCGA-43-A56V |
| TCGA-66-2756 | TCGA-63-A5MM | TCGA-77-6843 | TCGA-77-A5GH | TCGA-85-A4QQ | TCGA-46-3765 |
| TCGA-66-2757 | TCGA-63-A5MN | TCGA-77-6844 | TCGA-79-5596 | TCGA-85-A4QR | TCGA-46-3766 |
| TCGA-66-2758 | TCGA-63-A5MP | TCGA-77-6845 | TCGA-85-6175 | TCGA-85-A50M | TCGA-46-3767 |
| TCGA-66-2759 | TCGA-63-A5MR | TCGA-77-7138 | TCGA-85-6560 | TCGA-85-A50Z | TCGA-46-3768 |
| TCGA-66-2763 | TCGA-63-A5MS | TCGA-77-7139 | TCGA-85-6561 | TCGA-85-A510 | TCGA-46-3769 |
| TCGA-66-2765 | TCGA-63-A5MT | TCGA-77-7140 | TCGA-85-6798 | TCGA-85-A511 | TCGA-46-6025 |
| TCGA-66-2766 | TCGA-63-A5MU | TCGA-77-7141 | TCGA-85-7696 | TCGA-85-A512 | TCGA-46-6026 |
| TCGA-66-2767 | TCGA-63-A5MV | TCGA-77-7142 | TCGA-85-7697 | TCGA-85-A513 | TCGA-51-4079 |
| TCGA-66-2768 | TCGA-63-A5MW | TCGA-77-7335 | TCGA-85-7698 | TCGA-85-A53L | TCGA-51-4080 |
| TCGA-66-2769 | TCGA-63-A5MY | TCGA-77-7337 | TCGA-85-7699 | TCGA-85-A5B5 | TCGA-51-4081 |
| TCGA-66-2770 | TCGA-66-2727 | TCGA-77-7338 | TCGA-85-7710 | TCGA-90-6837 | TCGA-51-6867 |
| TCGA-66-2771 | TCGA-66-2734 | TCGA-77-7463 | TCGA-85-7843 | TCGA-90-7766 | TCGA-52-7622 |
| TCGA-66-2773 | TCGA-66-2737 | TCGA-77-7465 | TCGA-85-7844 | TCGA-90-7767 | TCGA-52-7809 |
| TCGA-66-2777 | TCGA-66-2742 | TCGA-77-8007 | TCGA-85-7950 | TCGA-90-7769 | TCGA-52-7810 |
| TCGA-66-2778 | TCGA-66-2744 | TCGA-77-8008 | TCGA-85-8048 | TCGA-90-7964 | TCGA-52-7811 |
| TCGA-66-2780 | TCGA-66-2753 | TCGA-77-8009 | TCGA-85-8049 | TCGA-90-A4ED | TCGA-52-7812 |
| TCGA-66-2781 | TCGA-66-2754 | TCGA-77-8128 | TCGA-85-8052 | TCGA-90-A4EE | TCGA-56-1622 |
| TCGA-66-2782 | TCGA-98-8022 | TCGA-77-8130 | TCGA-85-8070 | TCGA-90-A59Q | TCGA-56-5897 |
| TCGA-66-2783 | TCGA-98-8023 | TCGA-77-8131 | TCGA-85-8071 | TCGA-92-7340 | TCGA-56-5898 |
| TCGA-66-2785 | TCGA-98-A538 | TCGA-77-8133 | TCGA-85-8072 | TCGA-92-7341 | TCGA-56-6545 |
| TCGA-66-2786 | TCGA-98-A539 | TCGA-77-8136 | TCGA-85-8276 | TCGA-92-8063 | TCGA-56-6546 |
| TCGA-66-2787 | TCGA-98-A53A | TCGA-77-8138 | TCGA-85-8277 | TCGA-92-8064 | TCGA-56-7221 |
| TCGA-66-2788 | TCGA-98-A53B | TCGA-77-8139 | TCGA-85-8287 | TCGA-92-8065 | TCGA-56-7222 |
| TCGA-66-2789 | TCGA-98-A53C | TCGA-77-8140 | TCGA-85-8288 | TCGA-94-7033 | TCGA-56-7223 |
| TCGA-66-2790 | TCGA-98-A53D | TCGA-77-8143 | TCGA-85-8350 | TCGA-94-7557 | TCGA-56-7579 |
| TCGA-66-2791 | TCGA-98-A53H | TCGA-77-8144 | TCGA-85-8351 | TCGA-94-7943 | TCGA-56-7580 |
| TCGA-66-2792 | TCGA-98-A53I | TCGA-77-8145 | TCGA-85-8352 | TCGA-94-8035 | TCGA-56-7582 |
| TCGA-66-2793 | TCGA-98-A53J | TCGA-77-8146 | TCGA-85-8353 | TCGA-94-8490 | TCGA-56-7730 |
| TCGA-66-2794 | TCGA-J1-A4AH | TCGA-77-8148 | TCGA-85-8354 | TCGA-94-8491 | TCGA-56-7731 |
| TCGA-66-2795 | TCGA-L3-A4E7 | TCGA-77-8150 | TCGA-85-8355 | TCGA-94-A4VJ | TCGA-56-7822 |
| TCGA-66-2800 | TCGA-L3-A524 | TCGA-77-8153 | TCGA-85-8479 | TCGA-94-A5I4 | TCGA-56-7823 |
| TCGA-68-7755 | TCGA-LA-A446 | TCGA-77-8154 | TCGA-85-8481 | TCGA-94-A5I6 | TCGA-56-8082 |
| TCGA-68-7756 | TCGA-LA-A7SW | TCGA-77-8156 | TCGA-85-8580 | TCGA-96-7544 | TCGA-56-8083 |
| TCGA-68-7757 | TCGA-MF-A522 | TCGA-77-A5FZ | TCGA-85-8582 | TCGA-96-7545 | TCGA-56-8201 |
| TCGA-68-8250 | TCGA-NK-A5CT | TCGA-77-A5G1 | TCGA-85-8584 | TCGA-96-8169 | TCGA-56-8304 |
| TCGA-68-8251 | TCGA-NK-A5CT | TCGA-77-A5G3 | TCGA-85-8664 | TCGA-96-8170 | TCGA-56-8305 |
| TCGA-68-A59I | TCGA-NK-A5CX | TCGA-77-A5G6 | TCGA-85-8666 | TCGA-96-A4JK | TCGA-56-8307 |
| TCGA-68-A59J | TCGA-NK-A5D1 | TCGA-77-A5G7 | TCGA-85-A4CL | TCGA-96-A4JL |  |
| TCGA-6A-AB49 | TCGA-NK-A5D1 | TCGA-77-A5G8 | TCGA-85-A4CN | TCGA-98-7454 |  |
| TCGA-70-6722 | TCGA-NK-A7XE | TCGA-77-A5GA | TCGA-85-A4JB | TCGA-98-8020 |  |
| TCGA-70-6723 | TCGA-98-8021 | TCGA-77-A5GB | TCGA-85-A4JC | TCGA-56-8308 |  |
