# Supplementary material for: Bioinformatic analysis revealing mitotic spindle assembly regulated NDC80 and MAD2L1 as prognostic biomarkers in non-small cell lung cancer development
Source: BMC Med Genomics. 2020 Aug 14;13:112. doi: 10.1186/s12920-020-00762-5 (PMC7437940; doi:10.1186/s12920-020-00762-5)
Supplement: Supplementary file 2 — Additional file 2. Supplementary Table 2 The TCGA patients barcode for 223 LUAD patients samples. [file 12920_2020_762_MOESM2_ESM.docx]

**Supplementary Table 2 The TCGA patients barcode for 223 LUAD patients samples**

| TCGA-75-5125 | TCGA-44-5643 | TCGA-05-4430 | TCGA-55-1592 | TCGA-55-7576 | TCGA-55-8092 |
| --- | --- | --- | --- | --- | --- |
| TCGA-62-A471 | TCGA-44-3917 | TCGA-05-4424 | TCGA-50-5045 | TCGA-44-A4SS | TCGA-97-7554 |
| TCGA-86-8358 | TCGA-05-4402 | TCGA-55-7995 | TCGA-95-7947 | TCGA-L9-A7SV | TCGA-44-8120 |
| TCGA-05-4434 | TCGA-69-7979 | TCGA-95-8494 | TCGA-55-8299 | TCGA-62-8399 | TCGA-4B-A93V |
| TCGA-05-4395 | TCGA-62-A46O | TCGA-44-2666 | TCGA-80-5608 | TCGA-49-AAR3 | TCGA-55-A48Y |
| TCGA-73-4668 | TCGA-05-4427 | TCGA-05-5425 | TCGA-49-6743 | TCGA-55-8208 | TCGA-78-7535 |
| TCGA-78-7536 | TCGA-86-A4JF | TCGA-69-7974 | TCGA-69-7760 | TCGA-86-8673 | TCGA-50-5932 |
| TCGA-50-5072 | TCGA-78-7154 | TCGA-62-8394 | TCGA-05-4244 | TCGA-55-A48Z | TCGA-55-7727 |
| TCGA-64-5774 | TCGA-49-4507 | TCGA-55-7903 | TCGA-05-4245 | TCGA-44-5644 | TCGA-44-A47B |
| TCGA-50-6591 | TCGA-62-8402 | TCGA-05-4382 | TCGA-MP-A4TK | TCGA-73-4662 | TCGA-44-A47F |
| TCGA-05-4398 | TCGA-64-1676 | TCGA-86-7713 | TCGA-69-7980 | TCGA-75-6205 | TCGA-50-5939 |
| TCGA-05-4418 | TCGA-44-2656 | TCGA-L9-A743 | TCGA-75-5126 | TCGA-05-4403 | TCGA-95-7948 |
| TCGA-44-7662 | TCGA-44-4112 | TCGA-78-7150 | TCGA-73-A9RS | TCGA-99-8032 | TCGA-05-4425 |
| TCGA-55-1594 | TCGA-99-8033 | TCGA-55-1596 | TCGA-MP-A4T4 | TCGA-55-A494 | TCGA-44-2655 |
| TCGA-55-1595 | TCGA-05-4422 | TCGA-50-6594 | TCGA-44-6775 | TCGA-67-3772 | TCGA-44-A47G |
| TCGA-38-4628 | TCGA-80-5611 | TCGA-44-7672 | TCGA-69-7978 | TCGA-44-7671 | TCGA-97-A4LX |
| TCGA-73-7499 | TCGA-50-6592 | TCGA-MP-A4TC | TCGA-78-7160 | TCGA-93-A4JN | TCGA-38-4627 |
| TCGA-53-A4EZ | TCGA-69-7973 | TCGA-86-7954 | TCGA-55-8204 | TCGA-64-5778 | TCGA-91-6835 |
| TCGA-86-8074 | TCGA-86-7711 | TCGA-91-6831 | TCGA-MP-A4SY | TCGA-35-5375 |  |
| TCGA-49-AAR9 | TCGA-05-4432 | TCGA-99-8025 | TCGA-MP-A4T2 | TCGA-86-8279 |  |
| TCGA-NJ-A4YP | TCGA-05-4390 | TCGA-64-1677 | TCGA-86-8075 | TCGA-80-5607 |  |
| TCGA-55-8205 | TCGA-50-6590 | TCGA-86-8073 | TCGA-86-8278 | TCGA-55-7914 |  |
| TCGA-64-5775 | TCGA-55-6975 | TCGA-MP-A4TI | TCGA-86-8055 | TCGA-78-7147 |  |
| TCGA-55-8085 | TCGA-49-6767 | TCGA-55-7911 | TCGA-55-8301 | TCGA-L9-A8F4 |  |
| TCGA-62-8398 | TCGA-75-6207 | TCGA-05-4389 | TCGA-86-6851 | TCGA-49-4488 |  |
| TCGA-73-4676 | TCGA-97-8176 | TCGA-L9-A5IP | TCGA-64-1679 | TCGA-55-8091 |  |
| TCGA-55-5899 | TCGA-78-8660 | TCGA-95-A4VN | TCGA-05-5420 | TCGA-97-7553 |  |
| TCGA-95-7944 | TCGA-78-7145 | TCGA-55-8094 | TCGA-78-8640 | TCGA-44-2668 |  |
| TCGA-55-7994 | TCGA-95-7567 | TCGA-44-3919 | TCGA-53-7626 | TCGA-55-6983 |  |
| TCGA-78-7220 | TCGA-78-7143 | TCGA-78-7166 | TCGA-50-5049 | TCGA-62-A46Y |  |
| TCGA-50-5946 | TCGA-MN-A4N5 | TCGA-MP-A4T8 | TCGA-67-6215 | TCGA-62-A46R |  |
| TCGA-75-6214 | TCGA-55-A4DF | TCGA-J2-A4AD | TCGA-MP-A4T7 | TCGA-71-8520 |  |
| TCGA-50-5066 | TCGA-49-4514 | TCGA-05-5429 | TCGA-55-8614 | TCGA-97-8179 |  |
| TCGA-97-8175 | TCGA-50-5068 | TCGA-50-5933 | TCGA-95-7043 | TCGA-97-8177 |  |
| TCGA-73-4659 | TCGA-93-A4JQ | TCGA-69-7761 | TCGA-44-8117 | TCGA-49-4487 |  |
| TCGA-55-8620 | TCGA-05-4426 | TCGA-78-7148 | TCGA-97-8171 | TCGA-67-3771 |  |
| TCGA-MP-A4SV | TCGA-55-8203 | TCGA-MN-A4N1 | TCGA-55-8302 | TCGA-78-7153 |  |
| TCGA-55-6987 | TCGA-44-7660 | TCGA-NJ-A55R | TCGA-93-A4JP | TCGA-44-2665 |  |
| TCGA-53-7624 | TCGA-MP-A4TA | TCGA-55-8090 | TCGA-55-8511 | TCGA-86-8280 |  |
| TCGA-44-3918 | TCGA-75-5122 | TCGA-69-A59K | TCGA-75-6211 | TCGA-50-7109 |  |
| TCGA-78-7159 | TCGA-49-AAQV | TCGA-95-8039 | TCGA-MP-A4TE | TCGA-L4-A4E5 |  |
